# The mediating role of sleep quality in the relationship between dispositional mindfulness and fatigue in Chinese nurses during the COVID-19 pandemic

**DOI:** 10.1186/s12912-023-01642-w

**Published:** 2023-12-11

**Authors:** Caijun Dai, Pinglang Hu, Feifan Yan, Xuejiao He, Weizhen Cheng, Lihua Yu, Achang Fang, Xiaoling Meng, Meiyang Lou, Youying Chen, Danli Chi, Huasu Zhou, Qiaoge Chen, Zhenhong Fang, Shuhong Ni, Qiqi Huang

**Affiliations:** 1 Department of Pulmonary and Critical Care Medicine, Jinhua Municipal Central Hospital, Jinhua, Zhejiang Province China; 2 Department of Neurology, The First Affiliated Hospital of Wenzhou Medical University, Wenzhou, China; 3 Neurological Intensive Care Unit, The First Affiliated Hospital of Wenzhou Medical University, Wenzhou, China; 4 Department of Nursing, Zhejiang Jinhua Guangfu tumor Hospital, Jinhua, Zhejiang Province China; 5 Department of Nursing, Jinhua Hospital of Traditional Chinese Medicine, Jinhua, Zhejiang Province China; 6 Department of Nursing, Jinhua Municipal People’s Hospital, Jinhua, Zhejiang Province China; 7 Department of Nursing, Pujiang County People’s Hospital, Jinhua, Zhejiang Province China; 8 Department of Nursing, Pan’an County People’s Hospital, Jinhua, Zhejiang Province China; 9 Department of Nursing, Jinhua City Fifth Hospital, Jinhua, Zhejiang Province China; 10 Department of Nursing, The Second Hospital of Pujiang, Jinhua, Zhejiang Province China; 11 Department of Nursing, Wucheng People’s Hospital, Jinhua, Zhejiang Province China; 12 Department of Nursing, Jinhua City Maternal and Child Health Care Hospital, Jinhua, Zhejiang Province China; 13 Department of Cardiac Care Unit, The First Affiliated Hospital of Wenzhou Medical University, Wenzhou, China; 14 Department of Nursing, Jinhua Municipal Central Hospital, Jinhua, Zhejiang Province China; 15 Department of Pediatrics, The First Affiliated Hospital of Wenzhou Medical University, Wenzhou, China

**Keywords:** Dispositional mindfulness, Fatigue, Sleep quality, COVID-19, Mediation effect

## Abstract

**Background:**

During the COVID-19 epidemic in China, clinical nurses are at an elevated risk of suffering fatigue. This research sought to investigate the correlation between dispositional mindfulness and fatigue among nurses, as well as the potential mediation role of sleep quality in this relationship.

**Methods:**

This online cross-sectional survey was performed from August to September 2022 to collect data from 2143 Chinese nurses after the re-emergence of COVID-19. The significance of the mediation effect was determined through a bootstrap approach with SPSS PROCESS macro.

**Results:**

Higher levels of dispositional mindfulness were significantly negatively related to fatigue (r = -0.518, *P* < 0.001) and sleep disturbance (r = -0.344, *P* < 0.001). Besides, insufficient sleep was associated with fatigue (r = 0.547, *P* < 0.001). Analyses of mediation revealed that sleep quality mediated the correlation of dispositional mindfulness to fatigue (β = -0.137, 95% Confidence Interval = [-0.156, -0.120]).

**Conclusions:**

In the post-COVID-19 pandemic era, Chinese nurses’ dispositional awareness was related to the reduction of fatigue, which was mediated by sleep quality. Intervention strategies and measures should be adapted to improve dispositional mindfulness and sleep quality to reduce fatigue in nurses during the pandemic.

## Introduction

The COVID-19 pandemic enters its third year, and with the effective prevention and control of the epidemic, China is currently in the stage of normalized epidemic prevention. Although the psychological state of the public is gradually recovering, there are still some sporadic outbreaks that would cause more serious psychological problems [1]. Faced with the re-emergence of the epidemic, stressful working environments, and extra epidemic prevention work, medical staff still suffer from physical fatigue and psychological burden [2]. Thus, it is essential to focus on the potential physical and psychological issues arising from COVID-19, which could offer valuable insights for effective intervention and prevention strategies.

As the largest workforce within healthcare systems, nurses are essential in the fight against the COVID-19 epidemic [3]. Confronted with unprecedented challenges brought about by the pandemic, such as overwhelming workload, extreme stress, severe lack of sleep quality, and risk of infection, nurses are experiencing both physical and mental distress [4–7]. Fatigue is described as a subjective sensation of being tired or lacking energy, including both physical and mental fatigue. Previous studies have shown that the prevalence of moderate-to-high fatigue levels ranged from 35.06 to 72.2% during the COVID-19 pandemic [4, 8]. Nurses’ fatigue could cause various physical symptoms and negative emotions, further affecting their health and work performance [9]. Thus, the fatigue of nurses is worthy of attention during this particular period, and it is critical to relieve nurses’ fatigue to improve healthcare quality.

Mindfulness refers to the awareness that arises when individuals focus attention on the present purposefully and without judgment [10]. Not only described as a construct that can be induced via practice but mindfulness has also been conceptualized as a state or as a trait, playing a significant role in fatigue [11–14]. A plethora of previous research has shown that mindfulness-based interventions (MBIs) could promote psychological health, and alleviate suffering from fatigue [15, 16]. Mindfulness has been considered a protective factor against the unprecedented psychological impact caused by the COVID-19 pandemic [17]. Individuals’ psychological states were shown to be less negatively impacted by COVID-19 when they had higher levels of dispositional mindfulness [18]. Additionally, a previous study has revealed an association between mindfulness and the quality of professional life for nurses during the COVID-19 outbreak [19]. However, there are few studies examining whether mindfulness (dispositional mindfulness) could be a protective factor against fatigue among nurses during the COVID-19 pandemic. Therefore, this study aimed to explore the relationship between mindfulness (dispositional mindfulness) and the occurrence of fatigue among nurses during the epidemic.

In addition, a correlation between mindfulness and the quality of nurses’ sleep has been reported [20]. During the post-epidemic of the anti-COVID-19 era, the overall prevalence of sleep disturbances was 44.0% in clinical nurses, which would cause various adverse outcomes, including higher mental workload, and more fatigue [6, 7, 21]. According to a previous study, nurses in the COVID-19 care units could benefit from the implementation of the mindfulness-based stress reduction program in enhancing their sleep [22]. However, little is known about the role of sleep quality in the relationship between mindfulness and fatigue among clinical registered nurses during the COVID-19 pandemic. We speculated that sleep quality may be a possible pathway for mindfulness to impact the fatigue of nurses during the COVID-19 pandemic.

The primary purpose of this research was to investigate the association between mindfulness and fatigue among nurses during COVID-19, with sleep quality serving as a mediator. Our results might help develop better intervention programs to relieve fatigue and improve the sleeping habits of nurses. Two hypotheses were proposed: [1] Mindfulness would have a significant direct effect on fatigue [2]. Sleep quality would play a mediating effect between mindfulness and fatigue.

## Materials and methods

### Study design and participants

This web-based cross-sectional study was implemented from August 2022 to September 2022 corresponding to the period after COVID-19 flared up again in Jinhua, Zhejiang Province. Nurses from 11 hospitals in Jinhua voluntarily answered the self-administered Chinese anonymous questionnaires via an online platform (www.wjx.com).

### Measures

Participant demographics included age, gender, marital status, education, professional title, and years of nursing experience, while fatigue, mindfulness, and sleep quality were evaluated through the Chinese versions of validated psychometric tools.

The Mindful Attention Awareness Scale (MAAS) contains 15 items, which are answered on a 6-point Likert-type scale ranging from 1 (“almost always”) to 6 (“almost never”). MAAS was used to assess dispositional mindfulness [23], with items such as “I could be experiencing some emotion and not be conscious of it until sometime later”. Higher total scores of MAAS indicate a greater propensity for daily mindfulness. The Chinese version of MAAS has possessed robust psychometric properties and is widely utilized in practice [20, 24, 25]. The Cronbach’s alpha coefficient in this study was 0.929.

The level of fatigue was measured by the 14-item Fatigue Scale (FS-14) [26], with a total score ranging from 0 to 14. The higher score signifies more severe fatigue. In addition, items 1–8 of this scale could represent physical fatigue, while items 9–14 could represent mental fatigue. Previous studies demonstrated that the Chinese version of FS-14 was applicable to Chinese people [27, 28]. In this study, Cronbach’s alpha for internal consistency of the scale was 0.840.

Pittsburgh Sleep Quality Index (PSQI) measures sleep quality and sleep disturbances over the past month [29]. It consists of 19 self-assessed items classified into seven components: habitual efficiency, sleep latency, sleep duration, sleep disturbances, daytime dysfunction, subjective sleep quality, and use of sleep medication. Total scores were weighted from 0 to 21 with higher scores indicating increasingly poor sleep quality. In this study, Cronbach’s alpha coefficient of the PSQI was 0.790.

### Statistical analyses

Descriptive analyses were performed on demographic characteristics, reporting the means and standard deviations of continuous variables as well as the frequency and percentage of categorized data, while the correlation analyses of mindfulness, sleep quality, and fatigue were also calculated. The internal consistency of the scale was determined by calculating Cronbach’s Alpha coefficient. As for data validity, we examined the common method bias by the Harman single-factor test [30].

The mediation model was tested through the PROCESS macro version 4.1 (www.processmacro.org/index.html) (Model 4) [31]. For the best test of the mediation effect, the bootstrapping procedure (5,000 resamples) to measure the indirect effect was carried out, with the significant mediating (indirect) effect established by a 95% confidence interval (CI) excluding zero. The mediation analysis was also controlled for age, gender, and education level. All the research data were analyzed by the IBM SPSS statistics 23.0. Two-tailed *p*-values < 0.05 were considered statistically significant.

## Results

### Common method bias testing

Since the study data were gathered through self-report questionnaires, which might result in common method deviations, the Harman single-factor analysis was performed to screen for the common method bias. There was no serious common method bias in this investigation, as the results showed 6 factors with eigenvalues greater than 1, and the interpretation rate of the first factor was 29.589% (less than the 40% critical standard).

### Demographic characteristics and preliminary correlation analyses

The questionnaire was effectively completed by 2143 nurses, as shown in Table 1. Of the total sample, 2075 females (96.80%) were female, 1059 (49.40%) were married, 1343 (62.67%) had a college degree or above, and 1031 (48.10%) obtained a junior technical title. Participants were 30.15 years old on average (SD = 7.70) and had 9.17 years of nursing experience on average (SD = 8.36).

**Table 1:** Characteristics of all investigated variables

Variables	*n* (%)	Mean	SD
Age		30.15	7.70
Years of nursing		9.17	8.36
Gender			
Female	2075 (96.80%)		
Male	68 (3.20%)		
Educational Level			
Junior college or under	800 (37.33%)		
Undergraduate	1334 (62.25%)		
Graduate or above	9 (0.42%)		
Marital Status			
Unmarried	1046 (48.80%)		
Married	1059 (49.40%)		
Other	38 (1.80%)		
Professional Title			
Junior	1031 (48.10%)		
Intermediate	868 (40.50%)		
Senior	244 (11.40%)		

Abbreviations: SD, Standard deviation

The Pearson correlations for key variables were represented in Table 2. Sleep quality (r = -0.344, *P* < 0.001) and fatigue (r = -0.518, *P* < 0.001) were significantly negatively correlated with mindfulness. There was also a significant relationship between fatigue and sleep disturbance (r = 0.547, *P* < 0.001). Additionally, a significant association was also found between mindfulness and different perspectives of fatigue (physical fatigue: r = -0.448, *P* < 0.001; mental fatigue: r = -0.469, *P* < 0.001).

**Table 2:** Correlations between key study variables

Variables	Mean (SD)	1	2	3
1. Mindfulness (MAAS)	4.19 (0.92)	1		
2. Fatigue (FS-14)	8.17 (3.70)	-0.518^***^	1	
3. Sleep Quality (PSQI)	7.59 (3.53)	-0.344^***^	0.547^***^	1

^***^P < 0.001

Abbreviations: SD, Standard deviation; MAAS, The Mindful Attention Awareness Scale; PSQI, Pittsburgh Sleep Quality Index; FS-14, The 14-item Fatigue Scale

### Mediating effect analysis

**Fig. 1:**
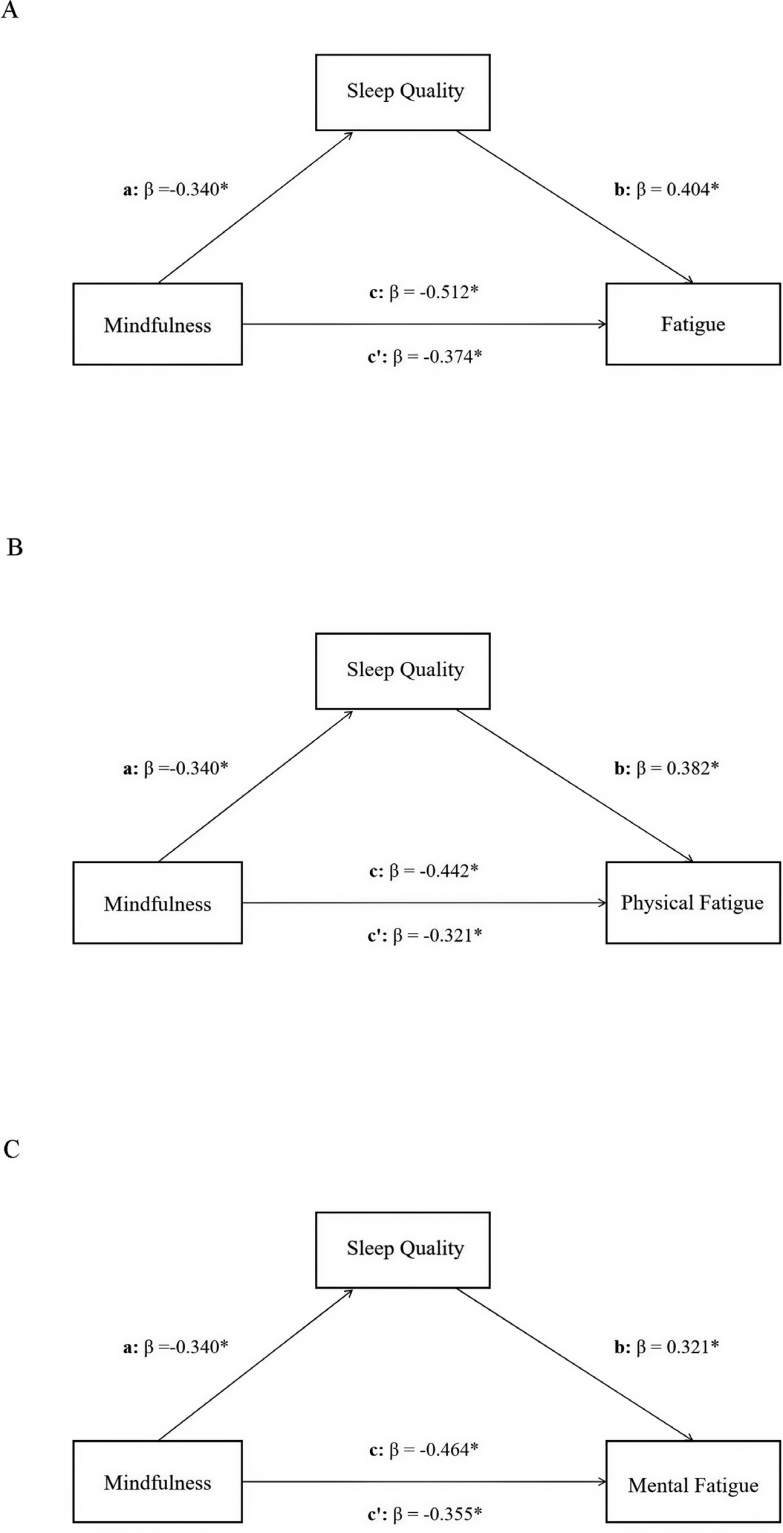
Relational model of dispositional mindfulness, sleep quality and fatigue (including different perspectives of fatigue). *Note*: (A) The model of sleep quality mediates the association between dispositional mindfulness and fatigue; (B) The model of sleep quality mediates the association between dispositional mindfulness and physical fatigue; (C) The model of sleep quality mediates the association between dispositional mindfulness and mental fatigue; c: total effect; c’: direct effect; β: standardized coefficient; ^*^P < 0.001

Following the preliminary results and correlations, sleep quality was examined as a potential mediator of mindfulness-fatigue associations through Model 4 in PROCESS. Age, gender, and education level were set as covariates. As shown in Table 3, mindfulness had a significant effect on nurses’ fatigue (β = -0.512, *p*-value < 0.001) and sleep quality (β = -0.340, *p*-value < 0.001). The direct effect was also statistically significant (β = -0.374, *p*-value < 0.001). By checking the bootstrapped 95% confidence interval, the significant indirect effect of sleep quality was identified in Table 4. This indicated that sleep quality mediated the relationship between mindfulness and fatigue. The mediation effect of sleep quality was also observed when fatigue was divided into two aspects (all *p*-values < 0.001) (Tables 3 and 4). Figure 1 illustrated the mediation model of dispositional mindfulness, sleep quality, and fatigue (including different perspectives of fatigue), along with standardized path coefficients. Besides, considering the cross-sectional design of this study and a possible bidirectional relationship between mindfulness and sleep quality, the reversed mediation model with mindfulness as a mediator was also analyzed, and the results were also significant (β = 0.129, bootstrap CI = 0.109,0.150).

**Table 3:** Mediating effect of Sleep Quality between Mindfulness and Fatigue (Physical Fatigue and Mental Fatigue)

Model	B	SE	β	t	*P*
Direct effect					
Mindfulness → Sleep Quality	-1.304	0.077	-0.340	-16.833	< 0.001
Mindfulness → Fatigue	-2.056	0.073	-0.512	-28.111	< 0.001
Mindfulness → Physical Fatigue	-1.172	0.051	-0.442	-23.141	< 0.001
Mindfulness → Mental Fatigue	-0.885	0.360	-0.464	-24.551	< 0.001
Indirect effects					
Mindfulness → Sleep Quality → Fatigue	-1.505	0.070	-0.374	-21.615	< 0.001
Mindfulness →Sleep Quality → Physical Fatigue	-0.828	0.049	-0.321	-16.787	< 0.001
Mindfulness → Sleep Quality → Mental Fatigue	-0.677	0.036	-0.355	-18.786	< 0.001

Abbreviations: SE, standard error; B, unstandardized coefficient; β, standardized coefficient

**Table 4:** Mediating model examination by bootstrap

Pathway	Effect*	SE	Boot LLCI	Boot ULCI
Mindfulness → Sleep Quality → Fatigue	-0.137	0.009	-0.156	-0.120
Mindfulness →Sleep Quality → Physical Fatigue	-0.130	0.009	-0.148	-0.111
Mindfulness → Sleep Quality → Mental Fatigue	-0.109	0.008	-0.126	-0.093

Abbreviations: SE, standard error; Boot LLCI, Bootstrap lower limit of confidence interval; Boot ULCI, Bootstrap upper limit of confidence interval; * Standardized indirect effect

## Discussion

The present study intensively investigated the relationship between dispositional mindfulness and fatigue in a large sample of 2134 Chinese nurses. Our findings revealed that mindfulness was considerably and inversely related to fatigue among nurses during the COVID-19 pandemic. When sleep quality was included, it mediated the association. Despite the high incidence of fatigue among nurses and the apparent impact of mindfulness-based interventions on fatigue, to our knowledge, this was the first study to explore the association between dispositional mindfulness and fatigue in nurses during the COVID-19 pandemic.

According to this analysis, mindfulness was negatively associated with nurses’ fatigue. The conclusion was in line with some studies that revealed that mindfulness was a protective factor against fatigue [12–14], and we further discussed that sleep quality could act on the mindfulness-fatigue connection. Mindfulness is the capacity to stay in the present moment and accept one’s experiences and emotions, which is related to enhanced psychological functioning and tolerance for unpleasant emotions and situations [32]. One of the previous studies has found that dispositional mindfulness could lessen fatigue through its effects on emotion regulation among nurses [13]. During the COVID-19 pandemic, higher levels of dispositional mindfulness could facilitate nonjudgmental awareness to increase the acceptance of COVID-19-related stressors, moderating the symptoms of anxiety and depression [17]. Thus, the higher the level of mindfulness in nurses, the less likely they would engage in fatigue when facing a stressful environment during the COVID-19 pandemic. In addition, this study also found the associations between mindfulness and different aspects of fatigue including physical and mental fatigue. This finding was interesting and further confirmed that there was a close relationship among mindfulness, body sensation, and mental health. Specifically, previous studies have demonstrated that mindfulness could alleviate the sense of physical fatigue by enhancing body awareness, reducing bodily tension, and improving self-regulation [23]. Besides, individuals with higher levels of mindfulness tend to demonstrate stronger emotional regulation, cognitive flexibility, and more positive mental health conditions, which may explain why they were less likely to be faced with mental fatigue [23]. These findings may provide more evidence of the effectiveness of mindfulness intervention in fatigue, and guide nursing managers to adopt strategies such as encouraging nurses to engage in short mindfulness meditation training [33], providing mindfulness-based mobile applications or online mindfulness-based programs [34, 35], or building a supportive community [36] to increase mindfulness levels of nurses during the epidemic period.

Consistent with prior research, our current results offered support for the correlation between fatigue and sleep quality in nurses during the COVID-19 outbreak [7, 37]. Heavy workloads and exposure to extreme stress limited nurses’ opportunity to adequately sleep after work hours, attributing to their feelings of fatigue and daytime dysfunction [6, 38]. In addition, the re-emergence of the epidemic would lead to the disruption of the recovered life, causing a negative psychological impact such as insomnia, anxiety, and depression on nurses, which would significantly influence their physical and mental fatigue [37–39]. Therefore, sleep quality-oriented intervention strategies and measures should be improved to effectively relieve fatigue among this population.

In addition, there was an association between mindfulness disposition and sleep quality among nurses. Previous studies have proved that mindfulness could protect nurses from sleep disturbance [20, 40]. Due to the beneficial impact of mindfulness in reducing stress-reactivity and increasing emotional balance, nurses with high mindfulness may appear more likely to stay with psychological equanimity in stressful contexts during the COVID-19 pandemic, which could be beneficial for sleep-related functioning [20]. Furthermore, it also has been demonstrated that the mindfulness-based stress reduction program could effectively enhance the sleep quality of nurses [22]. Further studies are still needed with more cases to explore the potential mechanisms of mindfulness on sleep quality and verify the validity of mindfulness-based interventions on nurses.

As this analysis was based on cross-sectional data, the results also indicated that the relationship between mindfulness and sleep quality could be reversed. This possible inverse hypothesis was also supported by another path analysis, which was similar to the previous study that nurses with satisfactory and sufficient sleep could predict next-day greater mindful attention [41]. A previous study has also mentioned that improved sleep quality may at times boost mindfulness [42]. Sleep may have an impact on individuals’ self-awareness and consciousness, circadian rhythms, brain function, and neurological health, which might influence the development of mindfulness [43, 44]. Thus, mindfulness also can be seen as a buffer with respect to the association between sleep quality and fatigue. Interventions and strategies such as encouragement of physical activity, cognitive behavioral therapy, provision of sleep education programs, and improvement of working and sleeping environment should also be considered for improving sleep quality in clinical nurses [45–47]. Future research that examines the relationship between sleep and mindfulness longitudinally might clarify the nature of their relationship. Nevertheless, the significant role of sleep quality and mindfulness in the fatigue of nurses should be recognized in any situation.

Some limitations of this study should be considered. First, the causality between variables could not be concluded in the current study due to the cross-sectional design. Besides, the dynamic psychological status of nurses could not be precisely reflected in the present study. A longitudinal follow-up study is still needed in the future. Second, the study did not distinguish whether the symptom was pre-existing or new due to the COVID-19 pandemic since the status of nurses before the outbreak was not evaluated, which might be a confounding factor. Third, the clinical nurses enrolled in this study were from partial areas of Jinhua, limiting the generalization of the findings. Fourth, the present research relied entirely on self-reporting, which would lead to bias and compromise the accuracy of the data, limiting the comprehensiveness of the current findings. Furthermore, the questionnaires used in this study were relatively simple. Further evaluation of patients’ anxiety, depression, and insomnia symptoms will be of high value. Fifth, we did not collect the data before and after the COVID-19 pandemic, which made it difficult for us to explore the distinction and how the distinction between during and not during the COVID-19 pandemic may impact the relationship between dispositional mindfulness and fatigue. Future studies were greatly needed to further explore this issue.

## Conclusions

In conclusion, the results identified dispositional mindfulness as a protective factor against the fatigue of nurses during the COVID-19 pandemic and revealed the role of sleep quality in mediating the association between mindfulness and fatigue. It is suggested that managers and nursing policymakers could provide and implement appropriate solutions to increase nurses’ mindfulness to directly or indirectly relieve their fatigue.

## Data Availability

The data are available from the corresponding author on reasonable request.
